# *Clonorchis sinensis* Infestation Promotes Three-Dimensional Aggregation and Invasion of Cholangiocarcinoma Cells

**DOI:** 10.1371/journal.pone.0110705

**Published:** 2014-10-23

**Authors:** Jihee Won, Jung-Won Ju, Sun Min Kim, Yoojin Shin, Seok Chung, Jhang Ho Pak

**Affiliations:** 1 School of Mechanical Engineering, Korea University, Seoul, Republic of Korea; 2 Division of Malaria & Parasitic Diseases, National Institute of Health, Korea Centers for Disease Control and Prevention, Osong, Republic of Korea; 3 Asan Institute for Life Sciences, University of Ulsan College of Medicine, Asan Medical Center, Seoul, Republic of Korea; INRS, Canada

## Abstract

Numerous experimental and epidemiological studies have demonstrated a correlation between *Clonorchis sinensis* (*C. sinensis*) infestation and cholangiocarcinoma (CCA). However, the role of *C. sinensis* in the increased invasiveness and proliferation involved in the malignancy of CCA has not been addressed yet. Here, we investigated the possibility that *C. sinensis* infestation promotes expression of focal and cell-cell adhesion proteins in CCA cells and secretion of matrix metalloproteinases (MMPs). Adhesion proteins help maintain cell aggregates, and MMPs promote the three-dimensional invasion of cells into the neighboring extracellular matrix (ECM). Using a novel microfluidic assay, we quantitatively addressed the role of excretory-secretory products (ESPs) gradients from *C. sinensis* in promoting the invasion of cells into the neighboring ECM.

## Introduction

Cholangiocarcinoma (CCA) is a disastrous, highly lethal malignancy of the biliary tract whose poor prognosis reflects difficulties in making an early diagnosis and a lack of effective therapy [1]–[3]. Risk factors for CCA are considered to include primary sclerosing cholangitis, fibropolycystic liver disease, intrahepatic biliary stones, chemical carcinogen exposure, viral hepatitis, and parasitic infestation. Many experimental and epidemiological studies have identified a strong correlation between liver fluke infestation and CCA [4]–[6]. Asian countries, including Thailand, Vietnam, Japan, Taiwan, Korea and China, have the highest incidence of CCA worldwide, mainly owing to chronic infection of bile ducts with *Clonorchis sinensis* (*C. sinensis*) and *Opisthorchis viverrini* through the intake of raw or undercooked freshwater fish harboring their metacercariae [7]. Clonorchiasis, an infection by *C. sinensis*, the third-most prevalent worm parasite in the world, can be fatal, largely because of the associated long-term complication of CCA. It is currently estimated that 15–20 million people are infected with *C. sinensis*, more than 200 million are at constant risk of infection [8], and 1.5–2.0 million show symptoms or complications of clonorchiasis. The International Agency for Research on Cancer (IARC) classified *C. sinensis* as a group I biological human carcinogen in 2009 [9], [10].

Although the precise mechanism linking the liver fluke with the development of CCA is not well understood, it has been proposed that chronic irritation and prolonged inflammation caused by direct contact with the worms and their excretory-secretory products (ESPs) provoke hyperplasia and adenomatous changes in the bile duct epithelium. During this process, persistent DNA damage and inhibition of DNA repair mechanisms occur, leading to apoptosis. These genetic lesions may be passed down to daughter cells through active cell proliferation, eventually leading to malignant transformation of normal cholangiocytes to CCA [7], [10]. During host infestation, liver fluke ESPs, an assortment of products (primarily proteins) that play important roles in host-parasite interactions, are continuously released into bile ducts and surrounding liver tissues. CCA cells exposed to liver fluke ESPs display diverse pathophysiological responses, including proliferation, apoptotic cell death, and inflammation [11]–[14]. For example, exposure of human CCA cells (HuCCT1) to *C. sinensis* ESPs has been shown to increase free radical generation through activation of NADPH oxidase, inducible nitric oxide synthase (iNOS) and xanthine oxidase, subsequently leading to nuclear factor-kappa B (NF-κB)-mediated inflammatory processes [15]. This latter study suggested that persistent oxidative stress during liver fluke infestation might disturb host cellular redox homeostasis, thereby creating vulnerabilities that predispose for the development of advanced hepatobiliary diseases such as inflammation-associated CCA. We also recently profiled changes in the transcriptomes and proteomes caused by exposure to *C. sinensis* ESPs [16], [17]. The genes/proteins differentially regulated by ESPs that were identified in these screens are involved in apoptotic modulation, carcinogenesis, metabolism, redox homeostasis and signal transduction, implying that ESPs contribute to multiple physiologic processes in host cells.

**Figure 1:**
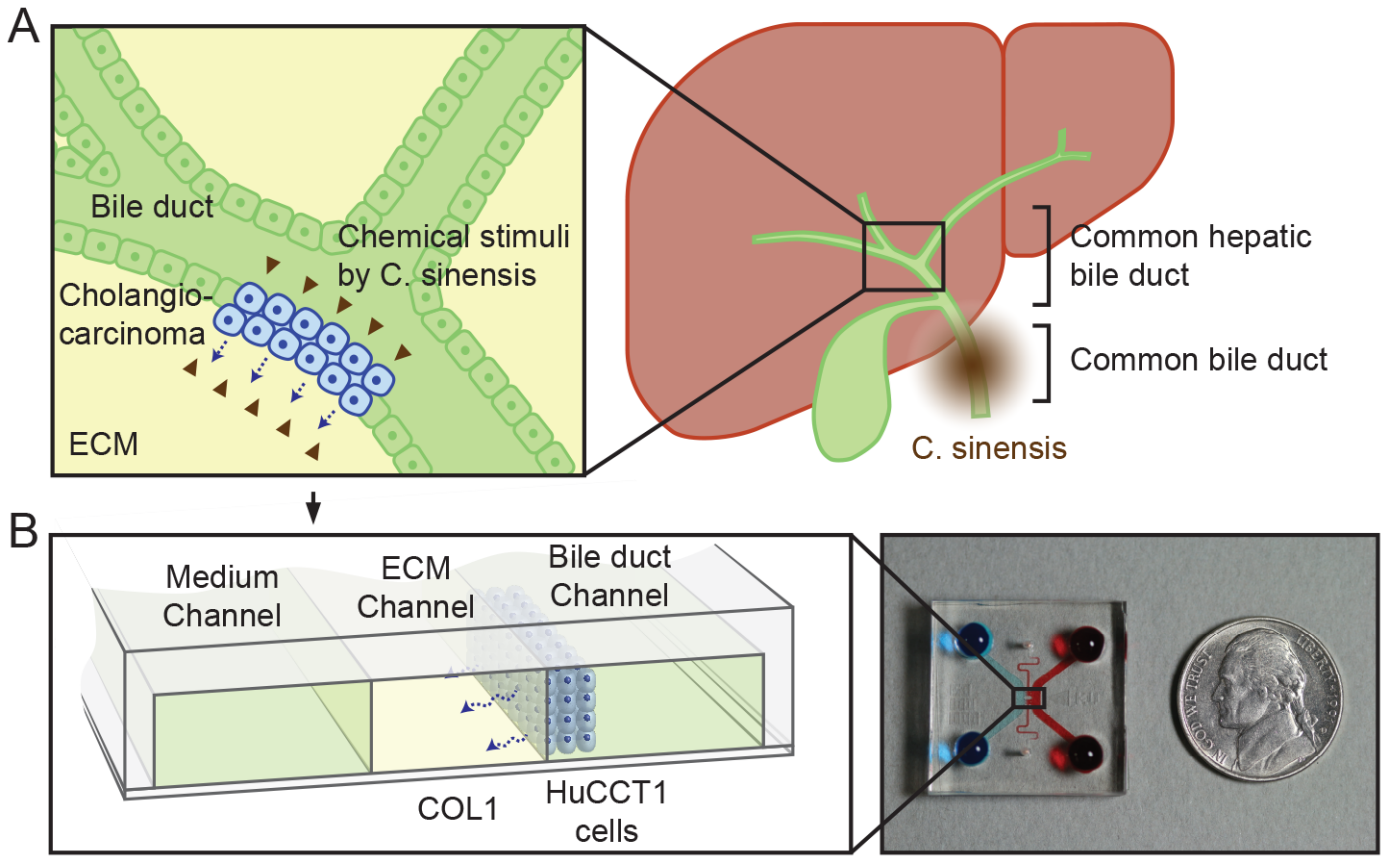
Depiction of hepatic bile duct and CCA. (A) Upon infection of the common bile duct, *C. sinensis* produces ESPs, which stimulate CCA. (B) CCA and ESPs stimuli can be simulated using a microfluidic platform, culturing HuCCT1 cells (in the bile duct channel) on a COL1 hydrogel incorporated in the ECM channel. The cultured HuCCT1 cells form aggregates on COL1 in the bile duct channel and invade into the COL1 in response to ESPs stimuli.

Can *C. sinensis* infestation also promote malignant CCA by increasing proliferation, invasion, and/or metastasis? More than 90% of CCAs are adenocarcinomas that are of epithelial origin [18]. The disease occurs within bile ducts, and migrates, invades and ultimately develops in peribiliary glands of the liver (**Fig. 1A**) [19]. The principal malignancy arises from the invasion and/or epithelial–mesenchymal transition of CCA into neighboring liver tissues; as a result, approximately 50% of patients with untreated disease die within 3–4 months of presentation [20]. Other cases of highly metastatic CCA have also been reported, for example toward lymph nodes [5], [17] and even the cerebellum [21]. However, to the best of our knowledge, there have been no reports on the increased mortality of CCA due to *C. sinensis* infestation.

**Figure 2:**
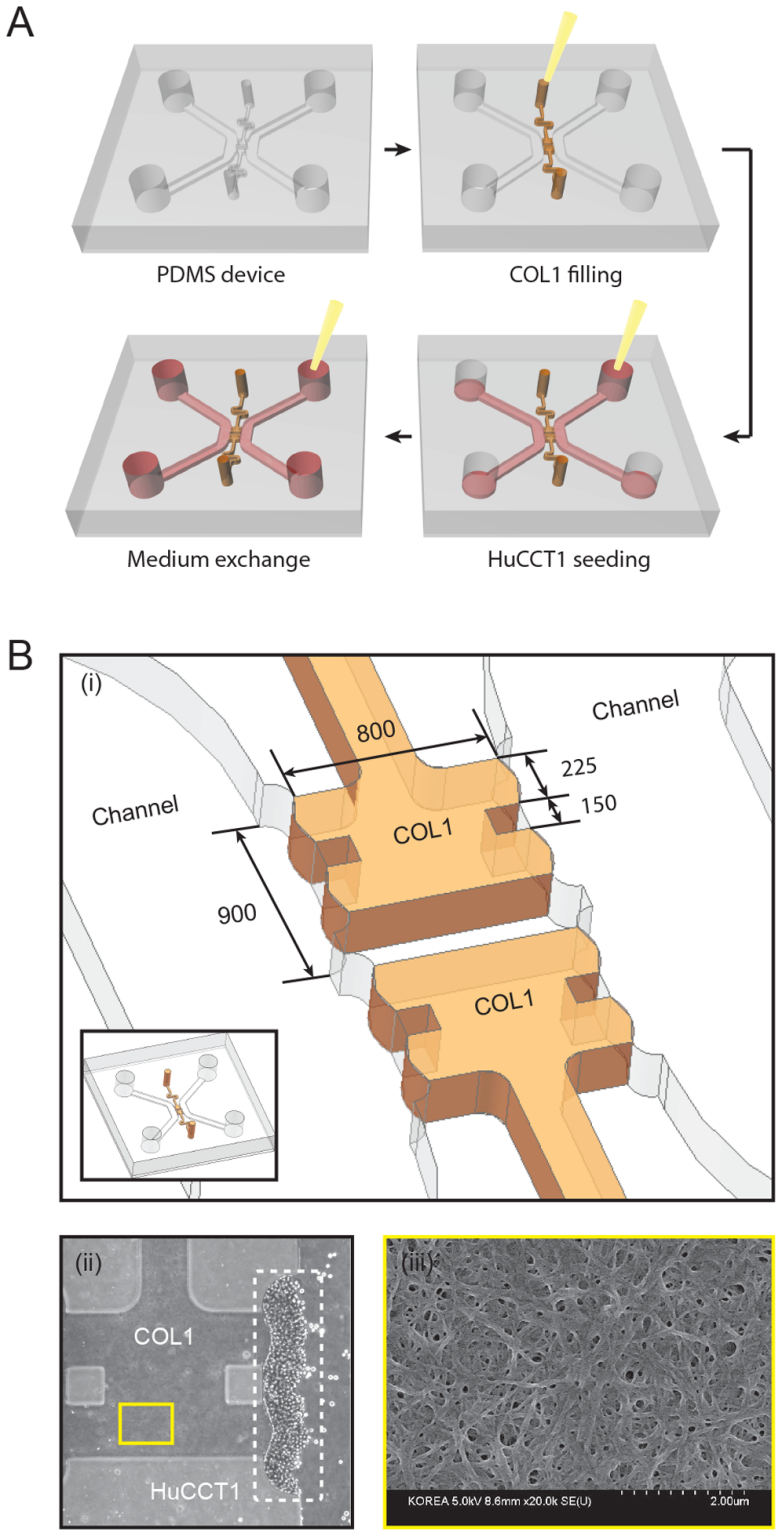
3D culture of HuCCT1 cells in a microfluidic device. (A) The PDMS device was bonded with a glass coverslip, and microchannels were coated with PDL. The gel port was filled with pre-polymerized COL1 solution and then the device was incubated at 37°C for 30 minutes. A cell suspension (1×10^6^ cells/ml) was injected into the bile duct channel. The cells were stacked onto the polymerized COL1 hydrogel by gravity by standing the device vertically for 2 hours. The medium was replaced with serum-free RPMI-1640 containing different concentrations of ESPs. (B) (i) Dimensions of the COL1 hydrogel and bowl-like structure that precisely fixes the number of cells on the COL1 hydrogel. (ii) Phase-contrast image of cells stacked on the collagen gel. Scale bar = 200 µm. (iii) SEM image of the fibrous structure of COL1.

During the past decade, a microfluidic three-dimensional (3D) cell culture assay system in which cells are cultured in microfluidic channels incorporating extracellular matrix (ECM)-mimicking hydrogels has been developed for modeling the *in vivo* cellular microenvironment. This system supplies precisely controlled conditions for the cells, including biochemical gradients, and cell–cell and cell–ECM interactions [22]. In the current study, we used this 3D cell culture system to analyze 3D growth and invasion of CCA cells into ECMs (**Figure 1** **and** **2**). The 3D hepatic ECM microenvironment was mimicked by filling the central channel of this system with type 1 collagen hydrogel (COL1) and allowing it to gel [23], before which HuCCT1 human cholangiocellular carcinoma cells, derived from a moderately differentiated adenocarcinoma with an epithelial-like morphology [24], were seeded (**Figure 2**). A scanning electron microscopic (SEM) image (**Fig. 2b**) shows the complex COL1 fibrous structure. HuCCT1 cells seeded onto the COL1 sidewall aggregated and formed a tumor mass in 1 to 2 days. Initial amount of HuCCT1 cells could be precisely fixed by the bowl-like structures around the COL1 sidewall (**Fig. 2b**). After the cells were seeded, the formation of 3D tumor masses and invasion of HuCCT1 cells were monitored daily by microscopy. In experiments, HuCCT1 cells were cultured in serum-free medium (control) or with ESPs stimuli, and the medium was refreshed daily. Details of assay preparation and experiments are summarized in the Materials Methods and in Supplementary Information (**Figure S1**).

## Materials and Methods

### Ethics statement

In preparation of C. sinensis ESPs, we sacrificed freshwater fish and New Zealand albino rabbits. Animal care and protocols were performed in accordance with institutional guidelines and were approved by the Animal Care and Use Committee of the Korea National Institute of Health. KCDC-Institutional Animal Care and Use Committee (KCDC-IACUC)/ethics committee reviewed and approved the ESPs preparation protocols (approval identification number; KCDC-003-11). Approximately fifty dead freshwater fish (*Pseudorasbora parva*) caught by local fishermen were collected to isolate the metacercariae in an endemic area of Korea (Namgang River, a branch of Nakdong River near Jinju city). No specific permissions were required for that location and activity.

### Preparation of *C. sinensis* ESPs

Whole flesh of the *Pseudorasbora parva* was digested with artificial gastric solution (0.6% pepsin in 0.7% HCl, pH 2.0) for 2 hours at 37°C. The digested content was then filtered through a 0.147-mm diameter sieve and washed thoroughly with 0.85% saline. *C. sinensis* metacercariae were collected under a stereoscopic microscope and stored at 4°C until infection. Three male New Zealand albino rabbits (10∼12 weeks old, ∼1.5–2 kg) were infected with ∼500 metacercariae via intragastric intubation and housed in individual cages at a constant temperature of 23°C in a 12 h light/dark cycle. The animals freely accessed a standard pellet diet and filtered tap water. At 12 weeks later, the rabbits were euthanized with a single intravenous injection of pentobarbital sodium (50 mg/kg). Adult worms were recovered from the bile ducts and washed several times with cold phosphate-buffered saline (PBS) to remove any host contaminants. Five fresh worms were cultured in 1 ml of prewarmed PBS containing antibiotic mixture and protease inhibitor cocktail (Sigma-Aldrich, St. Louis, MO, USA) for 3 hours at 37°C in a 5% CO_2_ environment. The culture fluid was then pooled, centrifuged, concentrated with a Centriprep YM-10 (Merck Millipore, Billerica, MA, USA), and filtered through a sterile 0.2-µm syringe membrane. The protein concentration of the ESPs was measured using DC Protein Assays (Bio-Rad, Hercules, CA, USA), and ESPs aliquots were stored at −80°C until use.

### Cell culture and ESPs treatment

The HuCCT1 human cholangiocarcinoma cell line (originally established by Miyagiwa et al. in 1989 [24]) was used as noted previously [15]–[17]. The cells were cultured in RPMI-1640 medium (HyClone, Logan, UT, USA) supplemented with 10% heat-inactivated fetal bovine serum (FBS; Gibco-BRL, Grand Island, NY, USA), 50 U/ml penicillin, and 50 µg/ml streptomycin at 37°C in a humidified 5% CO_2_ incubator. For treatment with ESPs, cells were seeded at ∼70% confluence on 60 mm culture dishes and grown for 24 hours under standard culture conditions. Cells were gradually deprived of serum by incubation in 1% FBS overnight, followed by incubation in serum-free medium for 3 hours. Cells were then treated with 800 ng/ml of ESPs for the indicated times.

### Preparation of the microfluidic assay system

Microfluidic devices were fabricated on a 4-inch silicon wafer patterned from SU-8-100 photoresist by UV photolithography. Polydimethylsiloxane (PDMS, Silgard 184; Dow Chemical, Midland, MI, USA) was cured on the SU-8–patterned wafer using a conventional soft lithography procedure. The PDMS device was punched using a 4-mm diameter dermal biopsy punch. The device was then sterilized twice at 120°C for 15 minutes followed by drying at 80°C for 6 hours. The sterilized device and glass coverslip (24×24 mm; Paul Marienfeld, Germany) were bonded using oxygen plasma (CUTE; Femtoscience, Hwaseong-si, Korea). The microchannels were filled with a 1-mg/ml solution of poly-D-lysine (PDL; Sigma-Aldrich, St. Louis, MO, USA) and incubated at 37°C for 3 hours. After washing the microchannels twice with sterilized deionized distilled water, the devices were dried at 80°C for 12 hours and stored at room temperature until use.

### 3D culture of HuCCT1 cells in the microfluidic assay system

A pre-polymerized type I collagen hydrogel solution was injected into the gel region of the microfluidic device. The hydrogel-filled assay was then incubated for 30 minutes at 37°C in a humidified atmosphere to allow complete gelation of the solution. The medium channels were filled with serum-free RPMI-1640 medium, and the device was kept at 37°C in a 5% CO_2_ incubator until use. A total of 60 µl of cell suspension (2×10^6^ cells/ml) was injected into a medium channel, and the device was placed vertically in the incubator for 2 hours to allow cells to attach to the hydrogel wall by gravity. The device was then positioned horizontally, and cells in the microfluidic assay system were cultured for the indicated times.

### Computation of ESPs transport

The concentration profile of ESPs was obtained from computational models using COMSOL Multiphysics 3.4 (COMSOL, Stockholm, Sweden) software. We assumed an average molecular weight of ESPs of 40 kDa, based on a previous assessment of the protein components of ESPs [25]. The diffusion coefficients of ESPs were set at 8×10^−11^ m^2^/s, 5.8×10^−11^ m^2^/s and 1.57×10^−12^ m^2^/s in cell growth medium, collagen gel and cell layer respectively, based on transport studies with 40 kDa dextran [26].

### Immunohistochemistry

Coverslips in 24-well plates were coated with a solution of rat tail collagen type I (50 µg/ml; BD Biosciences, San Jose, CA, USA) by incubating overnight at 4°C. After washing the coverslips with PBS, cells (2×10^4^) were seeded on the collagen-coated coverslips and grown overnight. Serum-starved cells were treated with 800 ng/ml of ESPs for 24 hours. After treatment, cells were fixed with 4% paraformaldehyde, permeabilized with 0.1% Trion X-100 in 0.1% sodium citrate, and incubated with monoclonal antibodies to vinculin (Sigma-Aldrich) or paxillin (BD Biosciences) overnight at 4°C. Subsequently, cells were incubated with a fluorescein isothiocyanate (FITC)-labeled goat antibody against mouse IgG (BETHYL Laboratories, Inc, Montgomery, TX, USA) and counterstained with Alexa Fluor 594-conjugated anti-phalloidin (Life Technologies, Carlsbad, CA, USA) for 10 minutes. The cells were air-dried, and coverslips were applied with mounting medium (Vector, Burlingame, CA, USA). Images were captured using a confocal laser-scanning microscope (LSM700; Carl Zeiss, Jena, Germany). Focal adhesion molecules on ESPs-treated cells were localized by first converting green fluorescent images to 16-bit gray scale images. Noise wasthen removed and background level was reduced using the threshold tool of ImageJ (US National Institutes of Health, Bethesda, MD, USA). As a result of these processes, green fluorescent points in the original images indicating the positions of focal adhesion molecules remained as dots, which were counted using the ‘Analyze Particles’ module in ImageJ.

### Quantitative real-time reverse-transcription polymerase chain reaction

Total RNA was extracted from ESPs-treated HuCCT-1 cells using an RNeasy mini kit (Qiagen, Valencia, CA, USA). Single-stranded cDNA was synthesized using the amfiRivert Reverse Transcription Kit (GenDEPOT, Barker, TX, USA) according to the manufacturer's instructions. qRT-PCR was performed on a Light Cycler 480 device (Roche Applied Science, Indianapolis, IN, USA) using a SYBR-Green PCR Master Mix (Applied Biosystems, CA, USA). Primer sequences for MMP1, −2, −9 and −13 were as follows: MMP1, 5′-CTGGAATTGGCCACAAAGTT-3′ (forward) and 5′-TCCTGCAGTTGAACCAGCTA-3′ (reverse); MMP2, 5′-GGAAAGCCAGGATCCATTTT-3′ (forward) and 5′-ATGCCGCCTTTAACTGGAG-3′ (reverse); MMP9: 5′-TTGGTCCACCTGGTTCAACT-3′ (forward) and 5′-ACGACGTCTTCCAGTACCGA-3′ (reverse); and MMP13, 5′-TCAGGAAACCAGGTCTGGAG-3′ (forward) and 5′-TGACGCGAACAATACGGTTA-3′ (reverse). The thermocycling conditions used were 30 cycles of 95°C for 20 seconds, 58°C for 30 seconds, and 72°C for 30 seconds. Each cDNA sample was run in triplicate. Endogenous 18 s rRNA was amplified in every plate as an internal control for sample variations. The mRNA level of each sample was normalized to that of 18 s rRNA, and the relative mRNA expression level of targets was presented as unit values of 2^[Ct(18 s rRNA)-Ct(each MMP)]^.

### Immunoblot analysis

Cells were washed with ice-cold PBS and then lysed with RIPA buffer (Sigma-Aldrich), followed by homogenization with a sonicator. After clearing lysates by centrifugation, total soluble protein (50 µg) was separated by sodium dodecyl sulfate-polyacrylamide gel electrophoresis (SDS-PAGE) on 10% gels. The proteins were transferred to a nitrocellulose membrane (GE Healthcare Biosciences, Uppsala, Sweden) and probed with primary antibodies against MMP1, −2, −9 or −13 (Abcam, Cambridge, MA, USA), followed by incubation with host-specific peroxidase-conjugated secondary antibodies. Immunoreactive proteins were detected with a West-Q chemiluminescent substrate kit (GenDEPOT) and quantified by densitometric scanning of the X-ray film with a Fluor-S Multi-imager (Bio-Rad). Blots were normalized with respect to protein loading by washing in BlotFresh Western Blot Stripping Reagent (SignaGen Laboratories, Gaithersburg, MD, USA) and reprobingwith a polyclonal antibody to glyceraldehyde-3-phosphate dehydrogenase (GAPDH; AbFrontier Co., Seoul, Korea).

## Results and Discussions

### ESPs treatment increases CCA cell tumor mass

**Figure 3:**
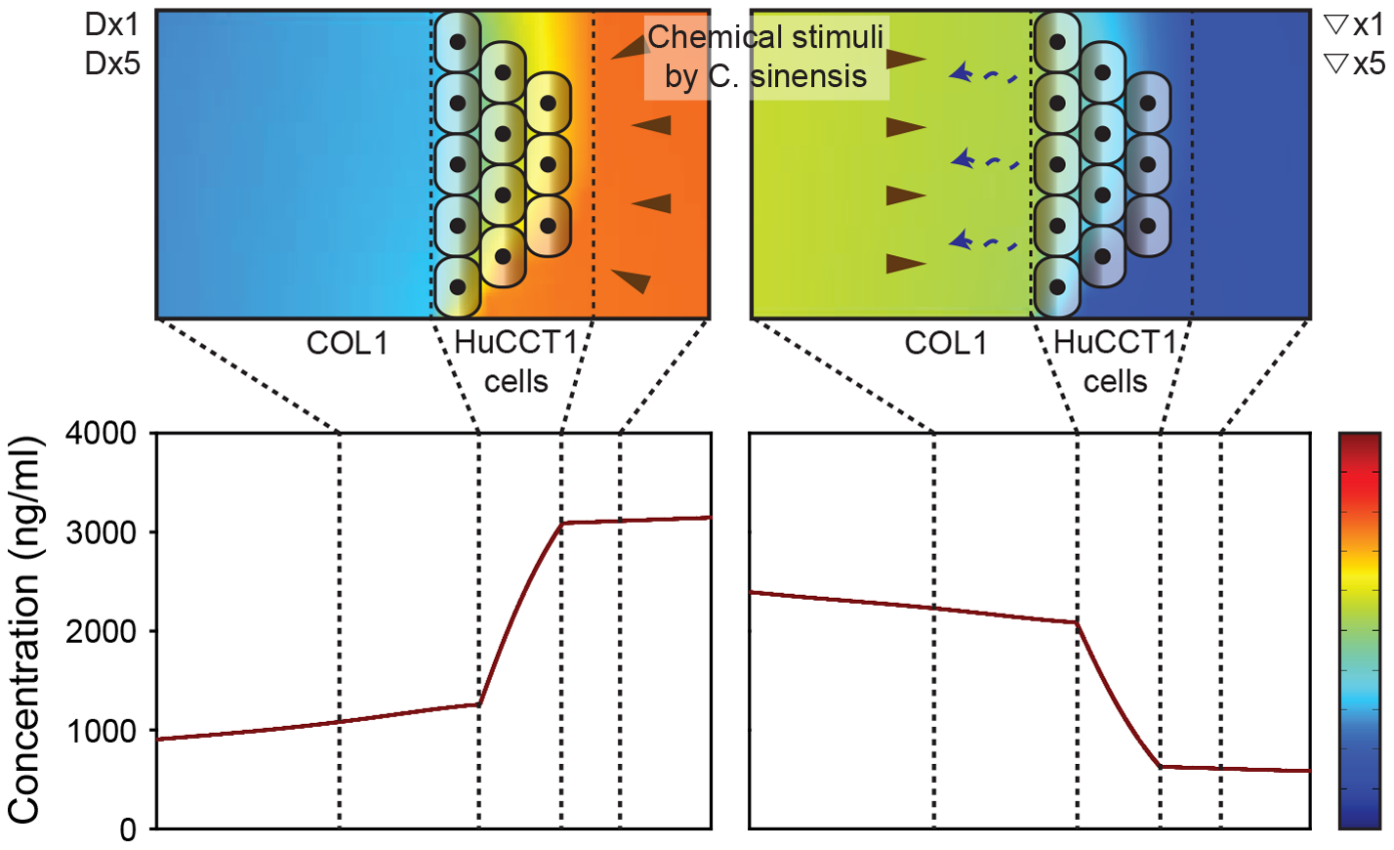
Concentration profiles of an ESPs-mimicking molecule in the COL1 hydrogel, media, and HuCCT1 cell aggregates. ESPs at a concentration of 4,000 ng/ml were delivered directly into the bile duct channel (left) or indirectly to form a gradient (right). Direct application resulted in a high concentration of ESPs in the bile duct channel in 24 hours (left). In the gradient application, ESPs formed a gradient and stimulated HuCCT1 cells attached onto the COL1 ECM in 24 hours (right).

The ESPs stimulus was applied directly to HuCCT1 cells by mixing ESPs with medium in the bile duct channel or as a gradient toward HuCCT1 cells by mixing ESPs with medium in the medium channel and allowing it to diffuse toward HuCCT1 cells (**Figure 3**). The ESPs concentration profile was evaluated using COMSOL software, assuming an average size of protein components in ESPs of 40 kDa (range, 30 to 50 kDa) [27]. In the direct application, ESPs accumulated in the medium outside of the HuCCT1 tumor mass. However, in the gradient application, ESPs accumulated in the ECM and directly stimulated HuCCT1 cells contacting the ECM. The concentration of ESPs applied directly in the bile duct channel was 800 ng/ml (Dx1) or 4,000 ng/ml (Dx5). Gradients toward HuCCT1 cells were formed by mixing ESPs with media in the medium channel at concentrations of 800 ng/ml (▽x1) or 4,000 ng/ml (▽×5). The gradient was formed in 30 minutes by molecular diffusion. Simulation showed that the gradient profile kept about 75% of initial concentration difference in 24 hours (**Figure 3**), and renewed by routine media refreshments in every 24 hours. HuCCT1 cells on the ECM blocked diffusion of ESPs and therefore stabilized their gradient profile. Accuracy of the simulated profile was already confirmed by previous reports [22], [28]–[30].

**Figure 4:**
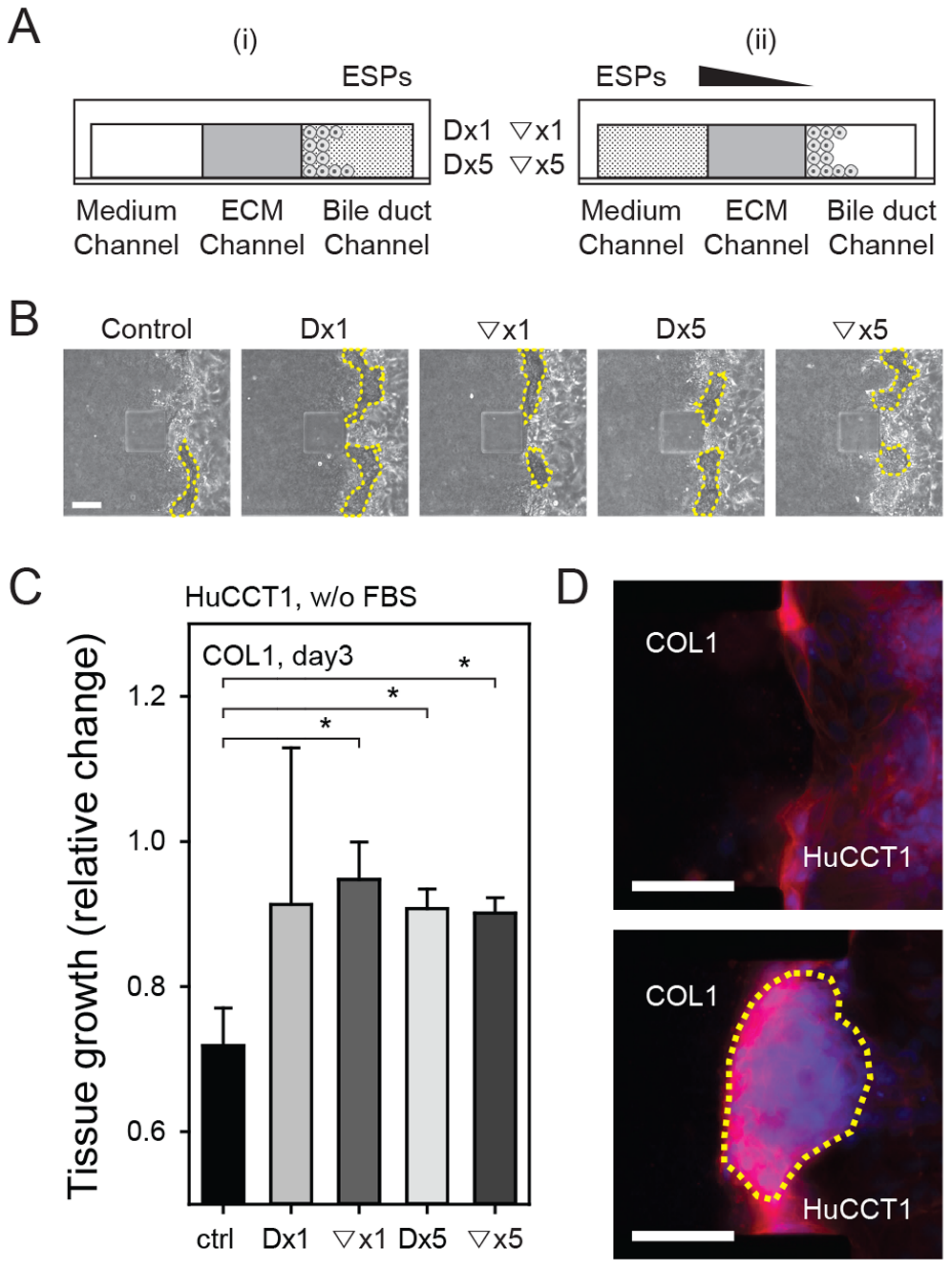
Formation of a HuCCT1 cell tumor mass in the microfluidic assay system. (A) ESPs exposure conditions: (i) direct stimulation (Dx1, Dx5), and (ii) indirect stimulation via gradient formation (▽x1, ▽x5). ×1 = 800 ng/ml; ×5 = 4,000 ng/ml. (B) Phase contrast images depicting growth of the HuCCT1 tumor mass. (C) Normalized growth of HuCCT1 cell tumors. *P<0.05 compared with the control. Significance was analyzed by Student's *t*-test. Error bars, ± SEM. (D) Fluorescence image of the HuCCT1 tumor mass (outlined in yellow dashed line) under untreated control (above) and Dx5 (below) conditions. Scale bars = 100 µm.

**Figure 5:**
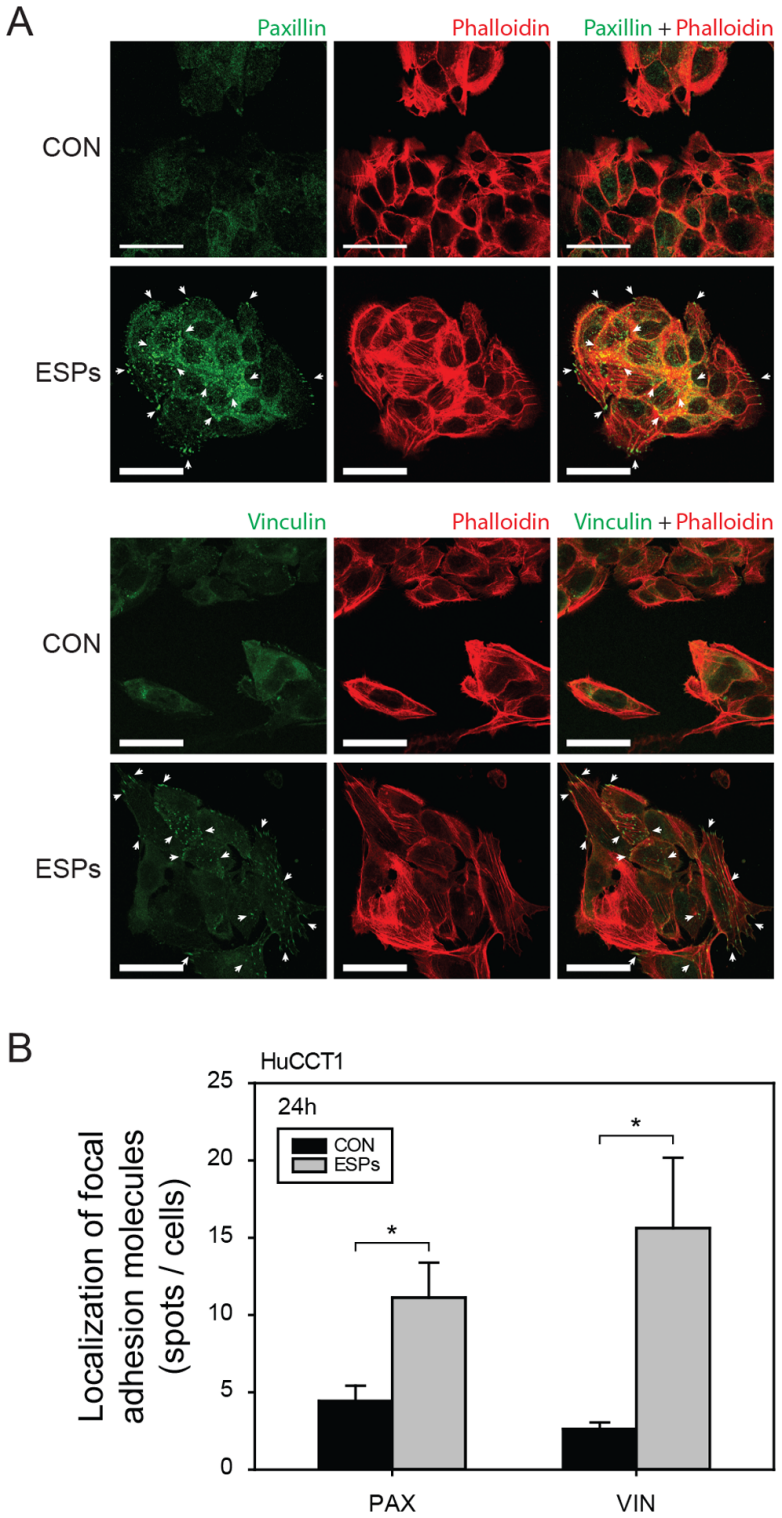
Expression and localization of focal adhesion molecules. (A) Expression of paxillin (above) and vinculin (below) in HuCCT1 cells under control and ESPs-stimulated (Dx1) conditions. Scale bars = 20 µm. (B) Quantification of localized paxillin (PAX) and vinculin (VIN) expression. *P<0.05 compared with the control. Significance was analyzed by Student's *t*-test. Error bars, ± SEM.

The size of HuCCT1 cell tumor masses on COL1 was monitored daily under serum-free (control) and ESPs-treated conditions (both gradient and direct applications) (**Fig. 4A**). The cells formed a tumor mass (**Fig. 4B and 4D**, yellow dashed line) on COL1. The size of the HuCCT1 tumor mass (projected area) was measured 3 days after the initial seeding, and was normalized to the size (projected area) on the first day. As shown in **Fig. 4C**, the HuCCT1 tumor mass tended to steadily decrease in size in both the absence and presence of ESPs. However, the presence of ESPs preserved tumor volume, maintaining more than 90% of the initial volume on day 3 compared with ∼70% for the control (**Fig. 4C**), an action that was essentially independent of the form (gradient or direct) or concentration (×1 or ×5) of the ESPs stimulus. This volume-preserving effect can be explained by an increase in the expression and localization of focal and cell-cell adhesion molecules (vinculin and paxillin) in cells treated with ESPs (direct application of 800 ng/ml, Dx1) (**Figure 5**). Vinculin is a membrane-cytoskeletal protein that is associated with both cell–ECM (integrin mediated focal adhesion) and cell–cell (cadherin mediated adhesion) junctions as well as tumor cell proliferation [31]–[33]. Paxillin is also a multi-domain adaptor protein with a major role in focal adhesion [34]. The increased expression and localization of both vinculin and paxillin in ESPs-treated cells would thus enable cells to maintain cell–ECM and cell–cell junctions and promote their proliferation, resulting in the conservation of tumor mass size.

### ESPs stimuli increased the invasiveness of CCA cells near the ECM

**Figure 6:**
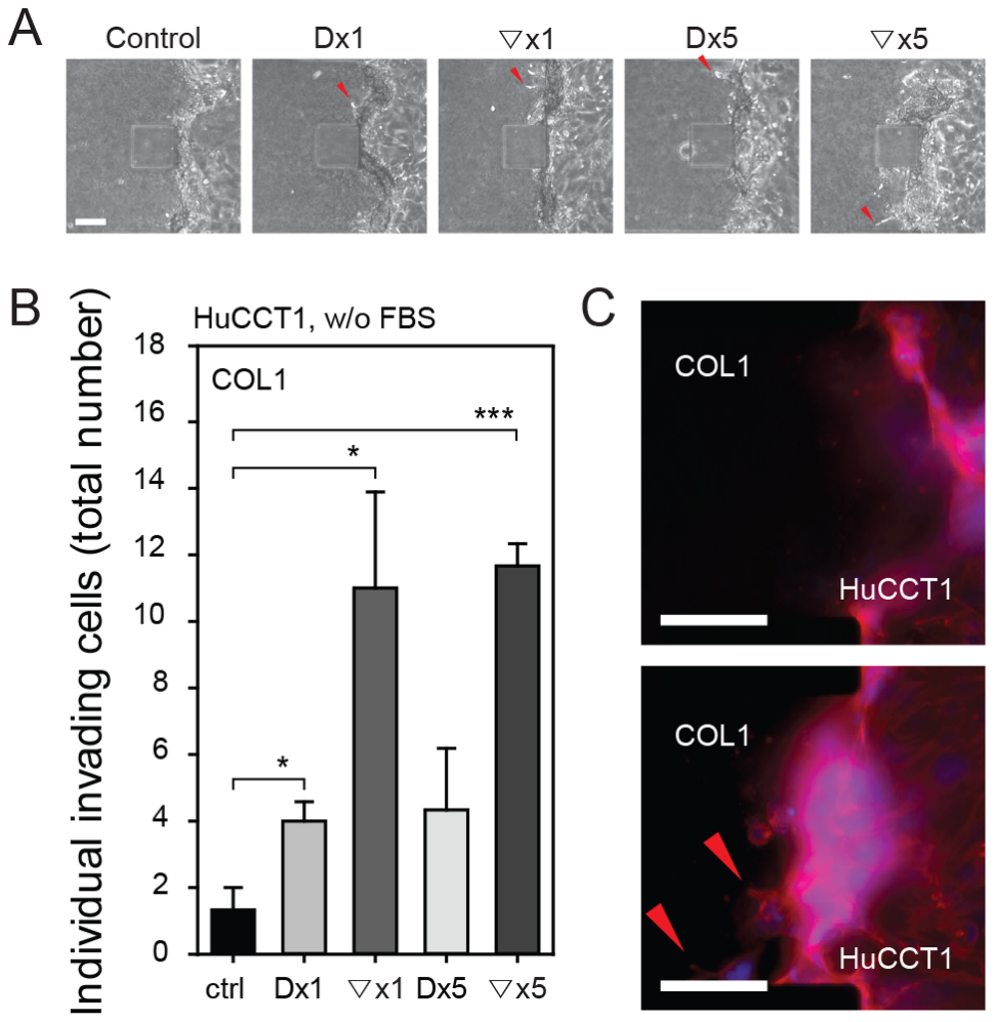
3D invasion of HuCCT1 cells into COL1 ECM over 6 days. (A) Phase-contrast image of protruding cells on day 3. (B) Summary data showing the total numbers of COL1-invading cells over 6 days. *P<0.05 and *** p<0.001 compared with the control. Significance was analyzed by Student's *t*-test. Error bars, ± SEM. (C) Immunofluorescence image of HuCCT1 cell penetration (red arrowheads) into COL1 under control (top) and Dx1 (bottom) conditions. Scale bars = 100 µm.

After tumor mass formation, HuCCT1 cells showed 3D invasion into COL1 under ESPs-stimulated conditions. The invasiveness of HuCCT1 cells was quantified by counting invading cells for 6 days. HuCCT1 cells have an epithelial-like morphology [24] and showed little invasion into COL1 under control condition (without ESPs). Both ESPs treatment paradigms promoted invasion of HuCCT1 cells into COL1, but the effect was much more dramatic when ESPs was applied in gradient form (▽x1 and ▽x5) (**Figure 6**). Both shallow (▽x1) and steep (▽x5) gradients of ESPs towards the HuCCT1 tumor mass induced a similar ∼10-fold increase in the number of HuCCT1cells invading into COL1.

**Figure 7:**
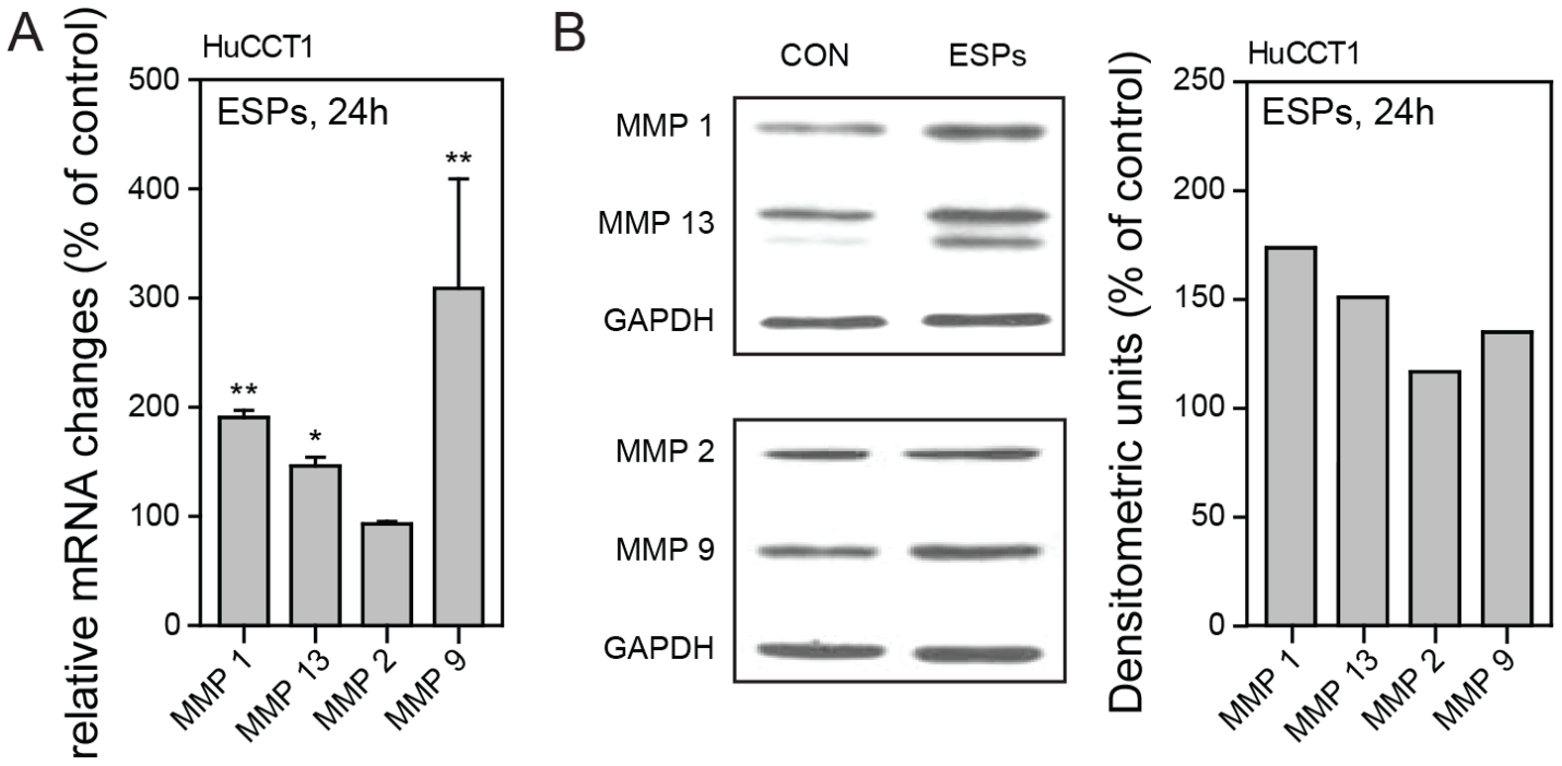
Effect of ESPs on MMP mRNA and protein expression. HuCCT1 cells were treated with 800 ng/ml ESPs or an equivalent volume of PBS, harvested after 24 hours, and analyzed for the expression of MMP1, −2, −9 and −13 mRNA and protein. (A) Expression of MMP1, −2, −9, and −13 mRNA normalized to 18 S rRNA was assessed by qRT-PCR. *P<0.05, ** p<0.01. Significance was analyzed by Student's *t*-test. Error bars, ± SEM. (B) Representative immunoblots of MMP1, −2, −9, and −13. The intensity of individual protein bands was determined by scanning densitometry and normalized to that of GAPDH. Data in graphs are expressed as a percentage of control values (in densitometric units).

Collagen turnover and ECM remodeling is modulated by a group of enzymes known as matrix metalloproteinases (MMPs) and their inhibitors, TIMPs (tissue inhibitors of MMPs), and the balance between MMPs and TIMPs is responsible for controlling the degradation of ECM proteins. MMPs play crucial roles in invasion and metastasis during tumor progression, and regulate signaling pathways that control cell growth, survival, invasion, inflammation, and angiogenesis [35], [36]. These observations prompted us to examine whether ESPs-induced invasion in 3D culture was associated with MMP activation. Changes in the expression of MMPs, including collagenases (MMP1, −8, −13) and gelatinases (MMP2, −9) were determined at the mRNA and protein level in cells treated with 800 ng/ml ESPs for 24 hours. Quantitative reverse transcription-polymerase chain reaction (qRT-PCR) analyses revealed a significant elevation of MMP1 (∼1.9-fold), MMP9 (∼3-fold), and MMP13 (∼1.5-fold) transcripts with ESPs treatment compared with untreated control (**Figure 7**). Consistent with these increases in mRNA, expression of the corresponding proteins was also increased ∼1.7-fold, ∼1.5, and 1.5-fold, respectively. These results indicate that MMP1, −9, and −13 expression were transcriptionally and translationally upregulated by ESPs, suggesting that MMPs secreted by ESPs-treated cells degrade COL1 fibers, enabling cells to more easily squeeze through and invade the pores among COL1 fibers. There was no evident change in MMP2 mRNA or protein expression in ESPs-treated cells, and neither MMP8 mRNA nor protein was detectable in HuCCT1 cells, as accessed by qRT-PCR and immunoblotting (data not shown). An animal model of *O.viverrini* infection has shown a close relationship between MMPs and TIMPs expression with peribiliary fibrosis. In particular, MMP9 expression was shown to be upregulated in the cytoplasm of inflammatory cells at the invasive front of the fibrous area, implying that MMPs and TIMPs play a role in the pathogenesis of opisthorchiasis [37].

Previous studies have reported increased invasion of CCA cells in matrigel invasion assays in a fibroblast co-culture system [38], [39]. We also found increased secretion of MMP9 in response to ESPs stimuli (**Figure 7**), which could explain HuCCT1 cell invasion into matrigel. However, hepatic ECM does not include basement membrane components, which are abundant in matrigel [23]. We were also unable to draw any definitive conclusions from our experiments showing 3D invasion into matrigel under ESPs-treated conditions (**Figure S2**), because the regulation of matrigel invasion by ESPs stimuli was strongly influenced by the large amount of protein in matrigel. COL1 better mimics hepatic ECM, suggesting a possible pathophysiological role of *C. sinensis* ESPs in CCA growth and invasion. Specifically, the increased expression of vinculin, paxillin, and MMPs induced by ESPs treatment could explain the increased invasion in response to ESPs stimuli in gradient form. The gradient increases ESPs concentration near the cells attached onto COL1 (**Figure 3**), selectively promoting secretion of MMPs and expression and localization of vinculin and paxillinin these cells. By promoting focal adhesion formation and enhancing the ability to degrade ECM, ESPs stimuli increase the invasion of HuCCT1 cells.

However, the dramatic increase in invasion even with a shallow gradient of ESPs (▽x1) cannot be fully explained, because the simulated values of ESPs concentration around cells attached to COL1 are similar in ▽×1 and D×5 conditions (data not shown). ESPs stimuli cause inflammation and damage HuCCT1 cells outside of the tumor mass [15], and the damaged cells near COL1 may promote migration of HuCCT1 cells in the tumor mass. This raises the potentially important issue of damage to the biliary tumor by ESPs and its role in increased malignancy. However, additional investigations using heterogeneous tissues of normal biliary cells and CCA cells will be required test this relationship.

## Conclusions

Using a novel microfluidic platform for monitoring 3D tumor mass formation and invasion into ECM, we describe a possible mechanism for the enhanced proliferation and invasion of CCA by *C. sinensis* infestation. ESPs treatment promoted the expression of the focal and cell–cell adhesion proteins, vinculin and paxillin, and increased the expression of MMPs (1, 9 and 13) in HuCCT1 cells. In tumor masses exposed to ESPs stimuli (direct stimulation), adhesion proteins helped cells in the tumor tightly adhere to each other and maintain their aggregate form. However, ESPs stimuli applied to cells outside of the tumor mass from the ECM side (gradient stimulation) promoted focal adhesion formation and enhanced the ability to degrade ECM. Thus, by promoting 3D invasion of HuCCT1 cells into neighboring ECMs, ESPs could enhance the mortality due to CCA.

## Supporting Information

Figure S1 **Fabrication and preparation of the PMDS microfluidic device.** Structures for molding microchannels were patterned onto a photoresist-coated silicon wafer using a photolithography method. The PDMS device containing micro-channels was made from the wafer by casting with a mixture of a PDMS base solution and curing agent (10∶1, w/w). The PDMS device and glass coverslip were irreversibly bonded using oxygen plasma. Immediately after bonding, 60 µl of PDL solution (1 mg/ml) was injected into microchannels, and the device was incubated at 37°C for 3 hours. After incubation, the PDL solution was removed by washing twice with sterilized deionized distilled water. The channels were dried by placing the device in an 80°C oven for 12 hours.
(TIF)

Figure S2 **3D invasion of HuCCT1 cells into matrigel (MAT) ECM over 6 days.** (A) Phase-contrast image of protruding cells on day 3. (B) Summary data showing the total numbers of MAT-invading cells over 6 days. Error bars, ± SEM. (C) Immunofluorescence image of HuCCT1 cell penetration (red arrowheads) into MAT under control (top) and Dx1 (bottom) conditions. Scale bars = 100 µm.
(TIF)

Checklist S1 (PDF)
