# Supplementary figures and images for: Clonorchis sinensis Infestation Promotes Three-Dimensional Aggregation and Invasion of Cholangiocarcinoma Cells
Source: PLoS One. 2014 Oct 23;9(10):e110705. doi: 10.1371/journal.pone.0110705 (PMC4207741; doi:10.1371/journal.pone.0110705)

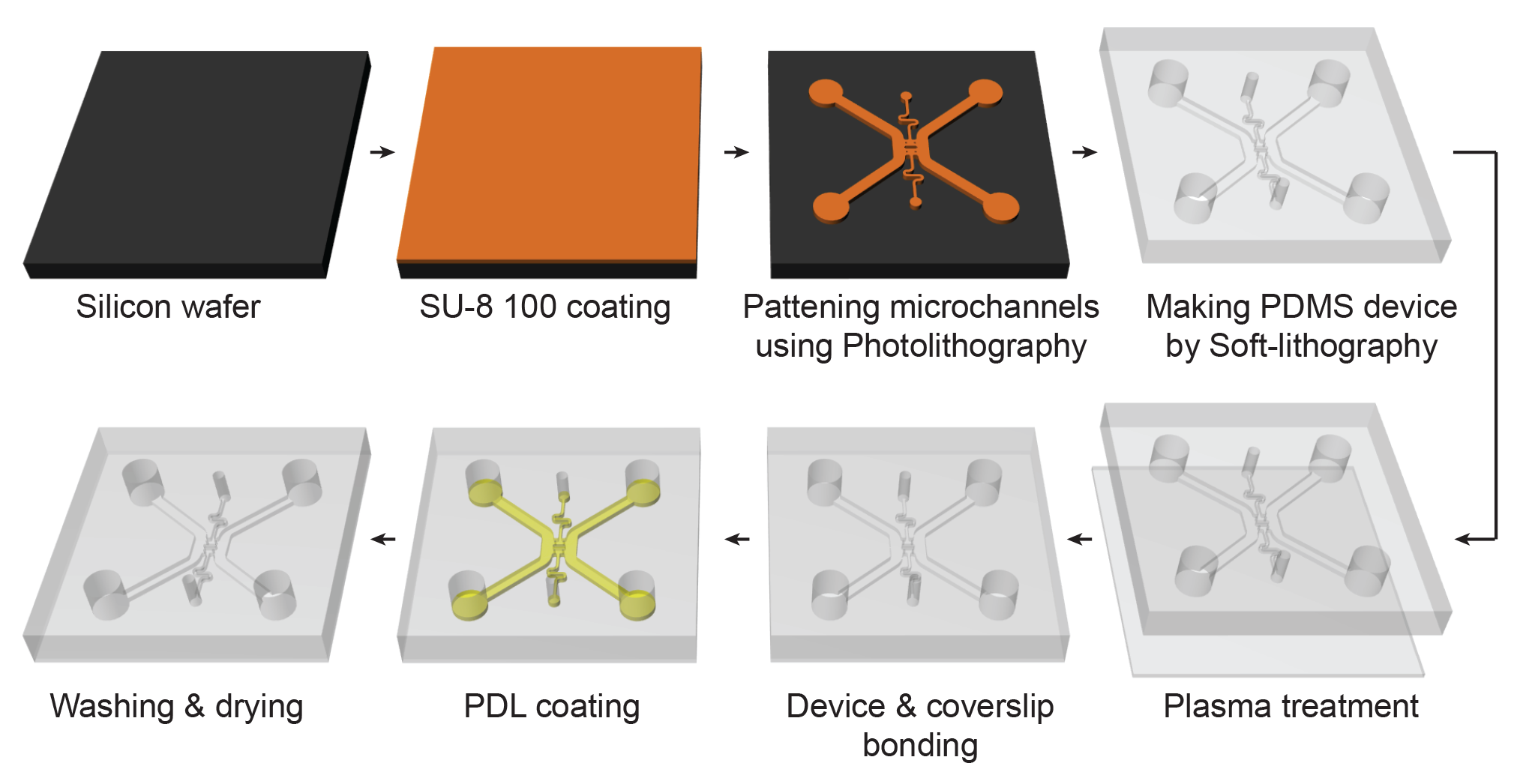

Supplement: Figure S1 — Fabrication and preparation of the PMDS microfluidic device. Structures for molding microchannels were patterned onto a photoresist-coated silicon wafer using a photolithography method. The PDMS device containing micro-channels was made from the wafer by casting with a mixture of a PDMS base solution and curing agent (10∶1, w/w). The PDMS device and glass coverslip were irreversibly bonded using oxygen plasma. Immediately after bonding, 60 µl of PDL solution (1 mg/ml) was injected into microchannels, and the device was incubated at 37°C for 3 hours. After incubation, the PDL solution was removed by washing twice with sterilized deionized distilled water. The channels were dried by placing the device in an 80°C oven for 12 hours. (TIF) [file pone.0110705.s001.tif]

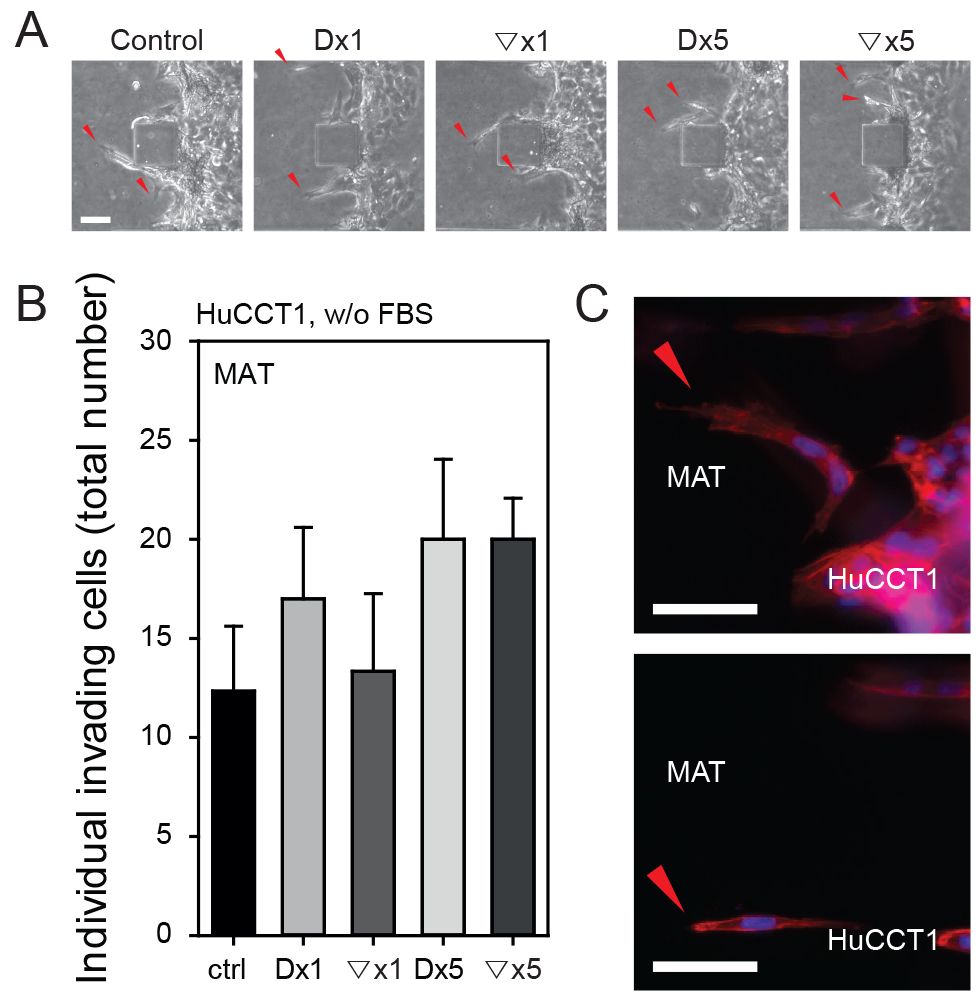

Supplement: Figure S2 — 3D invasion of HuCCT1 cells into matrigel (MAT) ECM over 6 days. (A) Phase-contrast image of protruding cells on day 3. (B) Summary data showing the total numbers of MAT-invading cells over 6 days. Error bars, ± SEM. (C) Immunofluorescence image of HuCCT1 cell penetration (red arrowheads) into MAT under control (top) and Dx1 (bottom) conditions. Scale bars = 100 µm. (TIF) [file pone.0110705.s002.tif]
